# Genome-scale insights into the metabolic versatility of *Limosilactobacillus reuteri*

**DOI:** 10.1186/s12896-021-00702-w

**Published:** 2021-07-30

**Authors:** Hao Luo, Peishun Li, Hao Wang, Stefan Roos, Boyang Ji, Jens Nielsen

**Affiliations:** 1 Department of Biology and Biological Engineering, Chalmers University of Technology, SE412 96 Gothenburg, Sweden; 2 Wallenberg Centre for Molecular and Translational Medicine, University of Gothenburg, SE405 30 Gothenburg, Sweden; 3 National Bioinformatics Infrastructure Sweden, Science for Life Laboratory, Chalmers University of Technology, SE412 96 Gothenburg, Sweden; 4 Department of Molecular Sciences, Uppsala BioCenter, Swedish University of Agricultural Sciences, SE750 07 Uppsala, Sweden; 5 BioInnovation Institute, Ole Måløes Vej 3, DK2200 Copenhagen N, Denmark

**Keywords:** *Limosilactobacillus reuteri*, *Lactobacillus*, Probiotic, Genome-scale metabolic model, Metabolic versatility, Core metabolism, Pan metabolism

## Abstract

**Background:**

*Limosilactobacillus reuteri* (earlier known as *Lactobacillus reuteri*) is a well-studied lactic acid bacterium, with some specific strains used as probiotics, that exists in different hosts such as human, pig, goat, mouse and rat, with multiple body sites such as the gastrointestinal tract, breast milk and mouth. Numerous studies have confirmed the beneficial effects of orally administered specific *L. reuteri* strains, such as preventing bone loss and promoting regulatory immune system development. *L. reuteri* ATCC PTA 6475 is a widely used strain that has been applied in the market as a probiotic due to its positive effects on the human host. Its health benefits may be due, in part, to the production of beneficial metabolites. Considering the strain-specific effects and genetic diversity of *L. reuteri* strains, we were interested to study the metabolic versatility of these strains.

**Results:**

In this study, we aimed to systematically investigate the metabolic features and diversities of *L. reuteri* strains by using genome-scale metabolic models (GEMs). The GEM of *L. reuteri* ATCC PTA 6475 was reconstructed with a template-based method and curated manually. The final GEM *i*HL622 of *L. reuteri* ATCC PTA 6475 contains 894 reactions and 726 metabolites linked to 622 metabolic genes, which can be used to simulate growth and amino acids utilization. Furthermore, we built GEMs for the other 35 *L. reuteri* strains from three types of hosts. The comparison of the *L. reuteri* GEMs identified potential metabolic products linked to the adaptation to the host.

**Conclusions:**

The GEM of *L. reuteri* ATCC PTA 6475 can be used to simulate metabolic capabilities and growth. The core and pan model of 35 *L. reuteri* strains shows metabolic capacity differences both between and within the host groups. The GEMs provide a reliable basis to investigate the metabolism of *L. reuteri* in detail and their potential benefits on the host.

**Supplementary Information:**

The online version contains supplementary material available at 10.1186/s12896-021-00702-w.

## Introduction

Probiotics are “live microorganisms that, when administered in adequate amounts, confer a health benefit on the host” and many of them are applied in disease treatment and food products [1, 2]. Some specific strains of *Limosilactobacillus reuteri*, previously known as *Lactobacillus reuteri* [3], are widely used as probiotics. *L. reuteri* are able to colonize in a wide variety of mammals and birds affecting the hosts’ health and metabolism. As a lactic acid bacterium that is generally recognized as a safe microorganism [4], some *L. reuteri* strains has been applied in a large variety of food products and food supplements throughout the world [1, 4], and also exploited as a potential cell factory [5]. With the shown beneficial properties of *Lactobacillus*/*Limosilactobacillus* strains, *L. reuteri* proved to have positive effects on several diseases such as improving symptoms of infantile colic, reducing diarrhea in children, preventing bone loss in the elderly and promoting regulatory immune system development and function [1, 6–10]. The *L. reuteri* ATCC PTA 6475 could prevent bone loss in a menopausal ovariectomized mouse model and were contented in chewable tablets as a dietary supplement [11–13]. Due to these advantages and possibilities, the interests of studying *L. reuteri* have significantly increased in recent years [14–16]*.*

The benefactions in metabolism may be due, in part, to the production of metabolites such as reuterin, histamine, vitamins and exopolysaccharide [17]. For example, histamine suppresses expression of tumor necrosis factor alpha and reuterin is known as an antimicrobial compound [18]. Interestingly, *L. reuteri* shows strain-specific effects on human health [17, 19]. Even among human-derived *L. reuteri* strains, the ability to reduce intestinal inflammation varies [19]. Recent studies have revealed the genetic diversity of *L. reuteri* strains [20, 21], which revealed that the diversification of *L. reuteri* strains could result from host-driven evolution, and some functional genes may be attributable to host-specific features [20, 21]. We were therefore interested to study the metabolism of individual *L. reuteri* strains in detail [8].

Genome-scale metabolic models (GEMs) are useful tools in metabolic engineering that could help us to understand the metabolism and physiology of the organism [22–24]. GEMs provide a way to integrate genome sequences, experimental data, and other types of data efficiently, as a platform to connect experimental data with internal metabolic mechanisms. The GEMs of several species from family *Lactobacillaceae*, such as *Lactobacillus plantarum* [25], *Lactobacillus casei* [26], *Lactobacillus reuteri* [5, 27] had been reconstructed and applied for simulation related to food fermentation, probiotics, and potential cell factory.

In this study, we reconstructed a comprehensive GEM for *L. reuteri* ATCC PTA 6475, namely *i*HL622, using a template-based method. To explore its metabolic characteristics as a probiotic strain, we simulated the growth with different carbon sources, amino acids usages and biosynthesis pathways of valuable products with experimental data. In order to explore the metabolic diversification of *L. reuteri* strains from different hosts, we further reconstructed metabolic networks for 35 *L. reuteri* strains. Comparison between *L. reuteri* strains revealed potential metabolic reactions related to host adaptation.

## Materials and methods

### Genome sequences

One of the genome sequences of *L. reuteri* ATCC PTA 6475 we used was provided by BioGaia, and the genome annotation was performed by the Prokka software [28] and the COG database. We also used two additional genomes of this strain sequenced by the Human Microbiome Project [29], which were collected from the NCBI database with accession numbers of NZ_ACGX00000000 and GCF_000159475.2 [29]. For the core and pan -models of the *L. reuteri* species, we collected 35 strains listed in a previous study [20], which could be downloaded from NCBI (Table S1)*.* The genome comparison was performed with BLASTP [30, 31] with following parameters: E value <=1E-10; bit score > = 100; percentage of positive scoring matches > = 45%. The sequences analysis was performed by the open-source package Biopython [32].

### Generation of *L. reuteri* GEMs

**Fig. 1:**
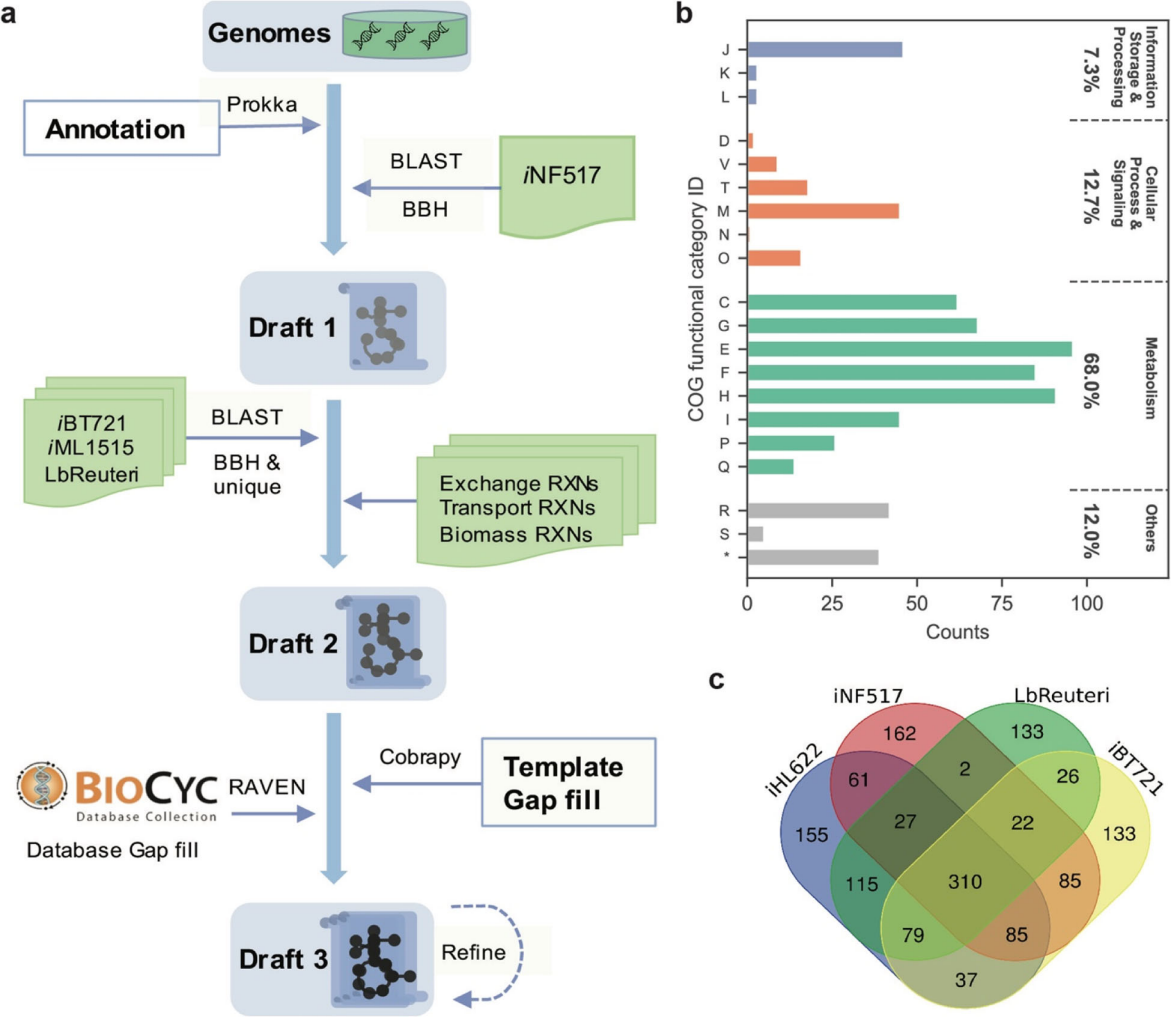
The *L. reuteri* ATCC PTA 6475 genome-scale metabolic reconstruction. (**a**) Template-based modeling pipeline. The *i*NF517 was employed as the primary template model and extracted ortholog genes and reactions based on bidirectional best hits (BBH) to generate the draft models. After comparing with LbReuteri, *i*ML1515 and *i*BT721, the exchange and transport reactions were added from the templates according to the transporter annotations and corresponding medium composition. The gap-filling was performed with COBRApy on the primary template model and used the MetaCyc database as a backup to improve the model performance. The GEM was also manually curated during the simulation and validation. (**b**) The COG functional distribution of genes in GEM. J, translation, ribosomal structure and biogenesis; K, transcription; L, replication, recombination and repair; D, cell cycle control, cell division, chromosome partitioning; V, defense mechanisms; T, signal transduction mechanisms; M, cell wall/membrane/envelope biogenesis; N, cell motility; O, posttranslational modification, protein turnover, chaperones; C, energy production and conversion; G, carbohydrate transport and metabolism; E, amino acid transport and metabolism; F, nucleotide transport and metabolism; H, coenzyme transport and metabolism; I, lipid transport and metabolism; P, inorganic ion transport and metabolism; Q, secondary metabolites biosynthesis, transport and catabolism; R, general function prediction only; S, function unknown; *, no COG categories. (**c**) The venn diagram of common and unique reactions in the four lactic acid bacterium models. *i*HL622 is the GEM of *L. reuteri* ATCC PTA 6475 in this study, *i*NF517, LbReuteri and *i*BT721 are the GEMs of *L. lactis* MG1363, *L. reuteri* JCM 1112 and *L. plantarum* WCFS1 separately

The GEM *i*HL622 of *L. reuteri* ATCC PTA 6475 was constructed by a template-based method with four templates, *i*NF517 (*Lactobacillus casei* MG1363) [26], LbReuteri (*L. reuteri* JCM 1112) [5, 27], *i*ML1515 (*Escherichia coli* MG1655, 33], and *i*BT721 (*Lactobacillus plantarum* WCFS1) [25]. As shown in Fig. 1a, a semi-automatic pipeline was developed for GEMs reconstruction. The *i*NF517 was employed as the main template to build the initial draft model, and orthologs genes were identified by the best bidirectional best hits (BBHs) from BLASTP results, with the parameters: E value <= 1E-10; bit score > = 100; percentage of positive scoring matches > = 45%. Then, the enzymes and associated reactions were integrated into the initial draft GEM by comparison against LbReuteri [5], *i*ML1515 [33] and *i*BT721 [25] one by one, this order takes into account homology and Memote scores. The exchange reactions and transport reactions were added according to the transporter annotations and corresponding medium composition. The default exchange reactions in our model corresponding a chemically defined medium with 111 mM glucose and serial amino acids like arginine that adopted from template model of LbReuteri. More medium conditions and description can be found in references [5, 27]. The gap-filling was performed with COBRApy [33–35] and used *i*NF517 as a template network. The resulting GEM was manually curated using the RAVEN [36] toolbox and reactions from the MetaCyc [37] database to improve the model performance. Since *L. reuteri* ATCC PTA 6475 is a well-studied probiotic, some potentially health-related metabolites could be produced such as lactate, acetate, ethanol, 1-propanol [38], and 1,3-propanediol [39], reuterin [40] (3-hydroxypropionaldehyde), histamine, vitamin B12 [41–43] (cobalamin) and vitamin B9 [42, 44] (folate). Therefore, missing reactions involving in these pathways were introduced into the draft GEM based on references and databases. For example, production of reuterin (3-hydroxypropionaldehyde) from glycerol is not annotated automatically but introduced manually.

After functional curation, the mass balance, charge balance and information annotation of the GEM were checked. To make the GEM recognized in other namespaces and connected with other databases, we generated annotations of EC number and the links to databases such as MetaCyc, KEGG and MetaNetX. Furthermore, MEMOTE [45] was applied to assess the GEM quality. With the proteome sequences of 35 strains, we performed the GEMs reconstruction for each strain as previously described [20].

The biomass reaction was adopted from the template models. The DNA coefficients were generated by the BOFdat toolbox [46]. The fraction weight of DNA was set to 0.031 g of 1 g biomass, and the detail coefficients of each nucleotide were set according to the DNA sequence GC content. The biomass content and composition of protein and lipid fractions were recalculated based on the LbReuteri model [5]. The code and model files could be found at a public GitHub repository (https://github.com/SysBioChalmers/Lactobacillus_reuteri_MM41A_GEM).

### Flux balance analysis

Growth capabilities in different mediums and essential amino acids validation were tested by flux balance analysis (FBA) [47]. We set the constraints of exchange reactions of medium and amino acids in the model with experimental substrate uptake rates and set the biomass reaction as the objective function to test the growth capability. During the testing of essential amino acids, D-glucose was selected as the sole carbon source and the uptake rate was set as 25 mmol/g DW/h from reference. When we tested the essential amino acids for growth, we set the uptake rate of 20 amino acids as 0 mmol / g [CDW] / h respectively. Growth rates under 1E-10 or infeasible results were considered as no growth. To test the synthesis capacities of products, we set secretion reaction of corresponding products as the object function to perform FBA. Secretory rates above zero pointed to that the model has corresponding synthesis capacity. The simulations were carried out in Python 3.7.9 with the COBRApy [33] 0.20.0 package and CPLEX optimizer 12.5.1 from IBM.

## Results and discussion

### The characteristics of *L. reuteri* ATCC PTA 6475 GEM

Three *L. reuteri* ATCC PTA 6475 genome sequences from different sources were collected. Two of them had been sequenced by the Human Microbiome Project [29] and the third one had been sequenced by BioGaia. Genome annotation of *L. reuteri* ATCC PTA 6475 from BioGaia yielded 2019 protein-encoding genes, 71 tRNA and 18 rRNA genes. Functional analysis based on clusters of orthologous groups (COG) classification showed that 80.5% of protein-encoding genes were mapped into COG categories. As shown in Fig. S1, 28.6% genes were related with metabolism and 26.3% genes associated with cellular process and signaling. The top three most abundant functional categories were ‘Mobilome: prophages, transposons’(X), ‘Translation, ribosomal structure and biogenesis’(J) and ‘Amino acid transport and metabolism’(E). Comparative genomic analysis shows that 1852 genes (93.17% on average) are shared between genomes from the three sources (Fig. S1b), while 102 protein-encoding genes were specific for strain from BioGaia. These three genome sequences got same complete value of 98.4% by BUSCO [48] analysis and the *L. reuteri* ATCC PTA 6475 genome sequence from BioGaia with only one contig was employed to reconstruct the GEM.

As shown in Fig. 1a, the GEM *i*HL662 was reconstructed by a template-based method. The initial draft model including 383 reactions and 465 metabolites was developed using *i*NF517 as template based on 763 BBHs. The metabolic genes and associated reactions mapping to the other three template models were also integrated into the draft model based on BBHs. The exchange reactions and transport reactions were added to enable nutrient uptake and by-product secretion, and gap-filling was performed to enable growth and by-product production. Furthermore, manual curations were conducted to remove potential errors in reactions or metabolites. Altogether, the final model *i*HL622 was obtained including 869 reactions and 713 metabolites with intracellular and extracellular components (Table 1), which is associated with 623 genes (30.8% of the genome) and 584 of them with COG categories (Fig. 1b). Compared with other published GEMs, there are 392 to 531 common reactions and 155 unique reactions in *i*HL622 (Table 1 and Fig. 1c). In addition, *i*HL622 included 31% more genes than the other three lactic acid bacterium templates models and there are 82% reactions in *i*HL622 associated with genes. MEMOTE analysis also showed the highest quality of *i*HL622 comparison against other GEMs.

**Table 1:** Model characteristics of *i*HL622 and comparison with template GEMs

Model	*i*HL622	*i*NF517	LbReuteri	*i*BT721	*i*ML1515
Organism	*L. reuteri* ATCC PTA 6475	*L.lactis* MG1363	*L. reuteri* JCM 1112	*L. plantarum* WCFS1	*E. coli* MG1655
Genes	2019	2339	1943	3063	4243
Included	622 (31%)	516 (22%)	530 (27%)	724 (24%)	1516 (36%)
Reactions	869	754	714	778	2712
Common with iHL622	869	483	531	392	509
With GPR^a^	709 (82%)	541 (72%)	606 (85%)	528 (68%)	2266 (86%)
Internal	644	530	507	538	1548
Transport	122	119	123	127	833
Exchange	103	105	84	113	331
Metabolites	713	650	660	662	1877
Unique	605	545	561	549	1071
Biomass consistency	1.00	0.83	-^b^	-^b^	1.00
MEMOTE Score	**80%**	60%	57%	38%	68%

a Gene-Protein-Reaction Associations

b Not applicable

### Prediction of *L. reuteri* ATCC PTA 6475 growth with different substrates

**Fig. 2:**
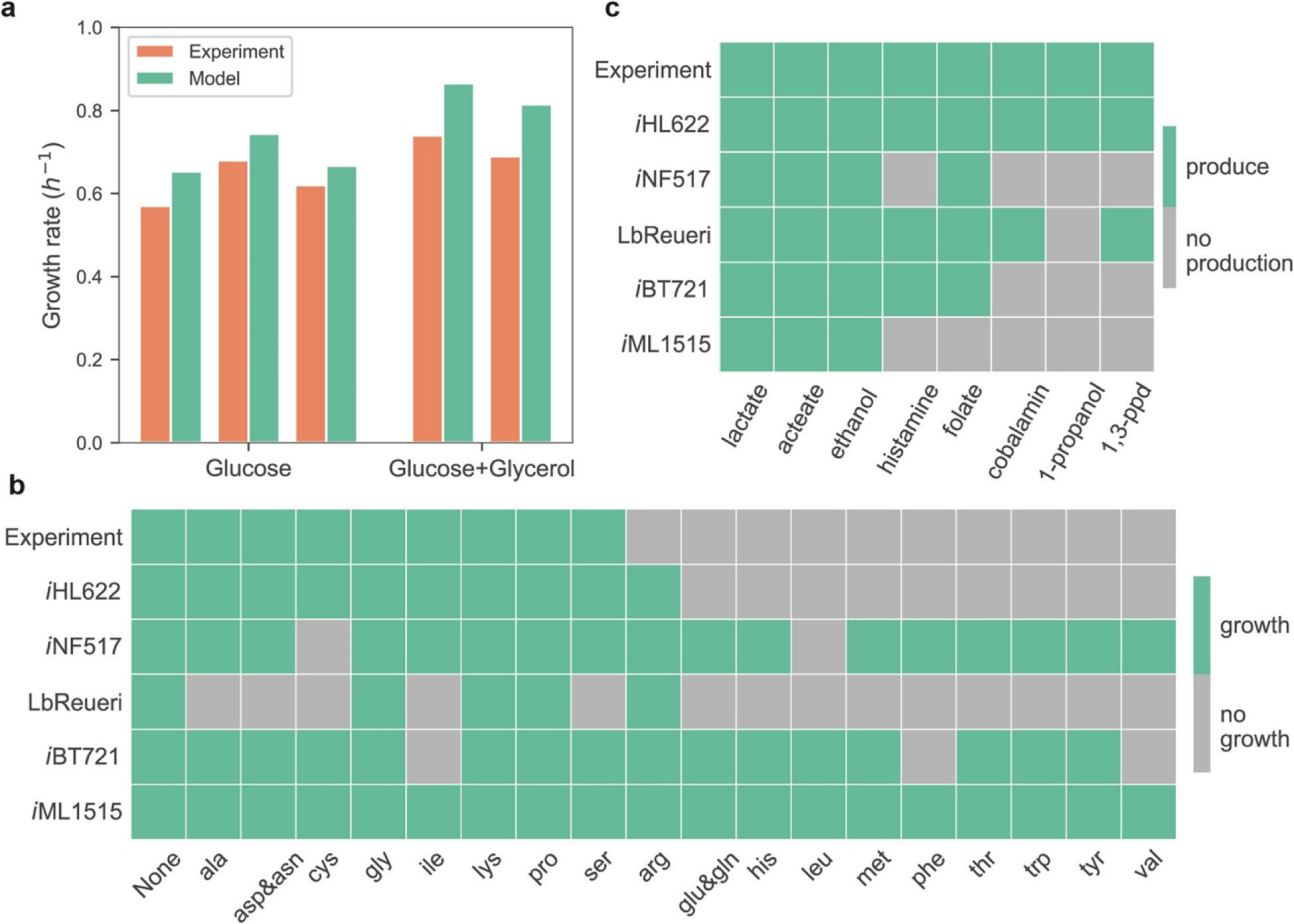
The predictions based on the *i*HL622 GEM. (**a**) Experimental and predicted growth rates. The experimental data for each dataset are shown in orange, and the prediction showing in green. (**b**) Growth capability under amino acid omitted medium. Experimental data are showed in the first row, while the predictions of single amino acid omission are shown in the rest rows. The growth showing in green and no growth showing in grey. (**c**) The predictions of representative metabolites. Eight products (lactate, acetate, ethanol, histamine, folate, cobalamin, 1-propanol and 1,3-propanediol) were predicted. Experimental data are showed in the first row, while the modeling results are shown in the rest rows. The produce showing in green and no productions showing in grey

*i*HL622 was employed to simulate growth of *L. reuteri* ATCC PTA 6475 under different growth conditions (Fig. 2). A previous study revealed that *L. reuteri* JCM1112, a highly similar strain of *L. reuteri* ATCC PTA 6475 [49, 50], grows faster with glycerol supplied and predominantly using the phosphoketolase (PK) pathway [5]. Therefore, *i*HL622 was used to simulate the growth capability with only glucose and with both glucose and glycerol by constraining the carbon sources uptake rates, and the exchange fluxes of other extracellular metabolites. Some studies have described the importance of *L. reuteri*’s glycolytic pathway and we also found that it could significantly affect the growth rate, so we added constrains of maximum flux of Embden-Meyerhof-Parnas (EMP) pathway to curate relevant pathways [5, 51]. Since both EMP and PK pathways exist in *L. reuteri,* the PK pathway should be dominant pathway, even the EMP pathway could provide more energy yield than the PK pathway [5, 51]*.* When reducing the EMP pathway flux with constraints on phosphofructokinase (PFK) and fructose-1,6-biphosphate aldolase (FBA) reactions [5], the growth rate was reduced significantly and close to the experimental data [5, 51, 52], which coincides with reports that the PK pathway shared the main carbon flux [5, 51]. In addition, the constraints of amino acids uptake rates and secretion rates of lactate and acetate were also added based on the experimental data. Altogether, the predicted specific growth rates are consistent with experimental observations [5]. The experimental growth rates are 0.751 ± 0.03 h^− 1^ with glycerol supplementation and 0.623 ± 0.04 h^− 1^ without glycerol, both are close to the values predicted by the model.

Moreover, *i*HL622 was used to predict the growth capability of *L. reuteri* ATCC PTA 6475 using amino acid as nitrogen sources (Fig. 2b). A previous study showed that eight non-essential amino acids (alanine, aspartate, cysteine, glycine, isoleucine, lysine, proline, serine) could be omitted from the growth medium and affected the growth rate and vitamin B12 production to different degrees [41]. The study also mentioned that the omission of serine reduced the specific growth rate about 80% whereas omission of other amino acids only caused ~ 13% reduction on average [41]. The qualitative predictions of single amino acid omission predictions are matching literature results except for arginine [41]. Since the uptake rate of amino acids were not mentioned in the reference, quantitative prediction was not performed. The mismatch between arginine predictions and experimental data may be due to the inherited characteristics from template GEMs and insufficient annotation of related enzymes.

Previous studies show that *L. reuteri* strains have the capacities to synthesize lactate, acetate, ethanol [53], histamine, folate [42], cobalamin [41–43], 1-propanol [54–56] and 1,3-propanediol [57]), which may be linked to the probiotic effects of *L. reuteri*. As shown in Fig. 2c, the biosynthesis capacities of *i*HL622 for these products were explored, and production of all these metabolites can be predicted correctly by *i*HL622.

### The metabolic features of *L. reuteri* ATCC PTA 6475

**Fig. 3:**
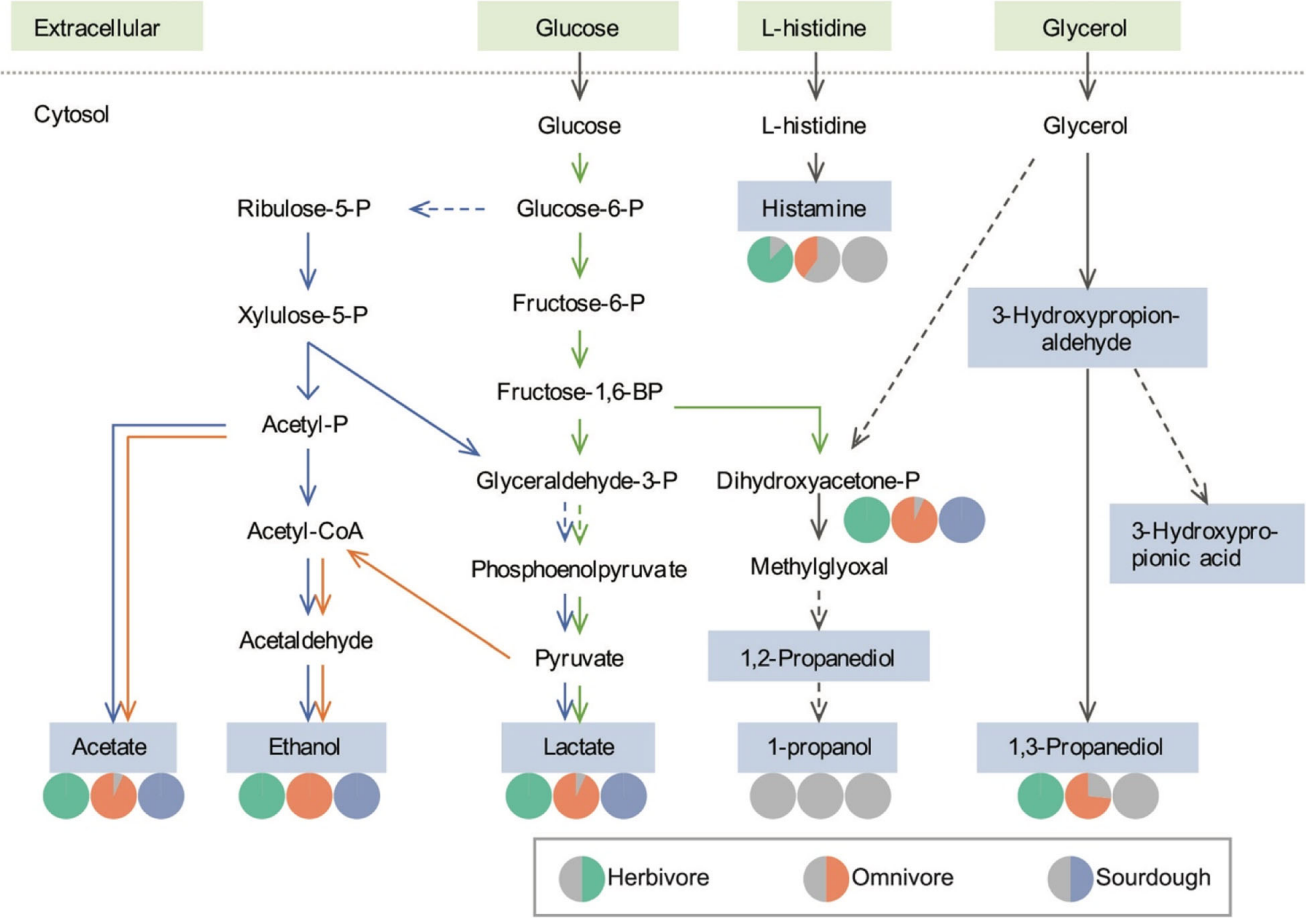
Overview of the metabolic pathways in *L. reuteri*. Green arrows indicate the phosphoketolase pathway (PKP); blue arrows indicate Emden-Meyerhof Parnas pathway (EMP); orange arrows indicate the extensions of EMP; and the dotted arrows indicate multiple enzymatic reactions. Green backgrounds indicate the extracellular metabolites and blue backgrounds indicate the *L. reuteri* products. The pie diagram under the metabolites shows the percentage of models in each group that could produce those corresponding metabolites. Green-grey pie diagram showing the group of herbivore-derived strains, orange-grey pie diagram showing the omnivore-derived group and blue-grey pie showing the sourdough-derived group

As a probiotic strain, *L. reuteri* ATCC PTA 6475 may affect host metabolism directly through secretion of metabolites that are influencing human cells. In order to investigate the health-promoting properties and metabolic features of *L. reuteri* ATCC PTA 6475, the main metabolic pathways were investigated tracked (Fig. 3). As mentioned before, carbohydrate metabolism mainly uses the PK pathway to produce lactate, acetate and ethanol, not EMP or EMP extensions even though they all appear in our model [5, 51, 52]. The PK pathway regulated by ribulose epimerase (MBLCLPDI_01299) and phosphoketolase (MBLCLPDI_01842) in the model. In food fermentation, lactate is usually the most important end-product of fermentation by lactobacilli, acetate and ethanol are main by-products, but the composition of the final end-products change dependent on growth conditions [4]. Due to its potential use as a biofuel, biosynthesis of 1-propanol has been extensively studied [54–56], and this metabolite can be produced from both glucose or glycerol. Here we focused on histamine and 3-HPA (reuterin), two potential beneficial metabolites synthesis genes and pathways. The histamine is a potential immunomodulatory factor that can modulate host mucosal immunity and suppresses pro-inflammatory tumor necrosis factor alpha production [18]. *L. reuteri* ATCC PTA 6475 have the histamine biosynthesis pathway and transporters that can convert L-histidine to histamine via histidine/histamine antiporter (hdcP, MBLCLPDI_01994), histidine decarboxylase pyruvoyl type A (hdcA, MBLCLPDI_01992), and hdcB (hdcB, MBLCLPDI_01991) [58]. The predicted histidine decarboxylase showed 95% identities against the histidine decarboxylase from conformed *L. reuteri* strains. 3-Hydroxypropionaldehyde (3-HPA) is the main component of reuterin that acts as a broad-spectrum antimicrobial substance and is an intermediate of the 1,3-propanediol synthesis pathway [57]. The 3-HPA production needs a one-step reaction from glycerol by the B12-dependent glycerol/diol dehydratase (PduC, PduD, PduE) [57, 59], which are encoded by the genes MBLCLPDI_01903, MBLCLPDI_01902 and MBLCLPDI_01901.

### Core and pan metabolism of *L. reuteri*

**Fig. 4:**
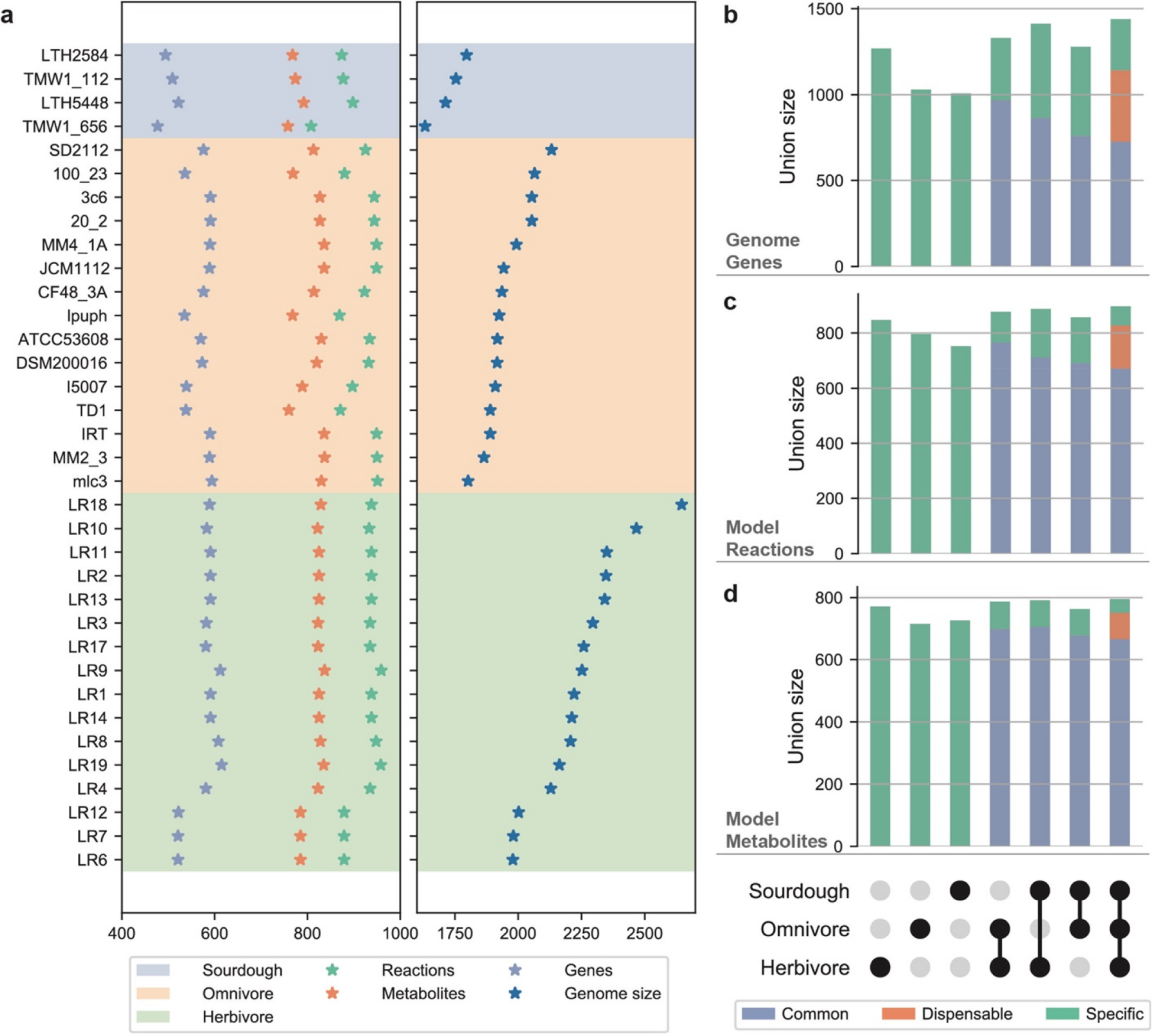
Characteristics of core- and pan-GEMs of 35 *L. reuteri* strains from different hosts. (**a**) Genome size shown in blue on the right, GEMs characteristics shown on the left. Green asterisks indicate the number of reactions, orange asterisks indicate the number of metabolites, and light blue asterisks indicate the number of genes in each GEM. These GEMs are grouped by host: herbivore, omnivore, and sourdough. The strains list in the y-axis are sorted in descending order by genome size in each group. (**b,c,d**) Upset plot of genes, reactions and metabolites between the pan-models of three groups. The total height of the bar indicates the union size of the corresponding group in the horizontal coordinate. In the final plotted bar, only appearing in one group is considered specific, appearing in all groups is considered common and appearing in two (between one and all) groups is considered dispensable. The common, dispensable and specific size from all combinations are shown

*Limosilactobacillus* species have been isolated from a wide range of sources. Recent genome sequencing of *Limosilactobacillus* species has provided basis to explore the metabolic diversity of *Limosilactobacillus* at the genome level [20, 21]. Some studies report that *L. reuteri* species from different ecological origins are closely associated with their living environment and genomic diversity [20, 21]. They also found some functional genes attributable to the host such as genes encoding cell surface proteins and active carbohydrate enzymes [20]. Here we analyzed the *L. reuteri* metabolism by metabolic modeling. The genome sequences of 35 *L. reuteri* strains were collected and used for GEMs reconstruction. These 35 strains can be classified into three distinct groups based on their corresponding host including herbivore, omnivore, and sourdough, with a distribution of 16, 15, and four strains into the three groups respectively. The genome size and GEMs characteristics are shown in Fig. 4a, with a genome size of 2058.3 ± 222.9 CDS and GEMs of 919.8 ± 35.0 reactions and 811.0 ± 25.7 metabolites linked to 567.1 ± 35.6 encoding genes. Here we found that the GEMs size is weakly correlated with genome size, the genome size is sorted in descending order (Fig. 4a, right) while none of the model characteristics correspond to this order (Fig. 4a, left).

Moreover, group-wised core and pan metabolic models were reconstructed. In the herbivore group, the GEMs have 929.2 ± 26.1 reactions and 818.8 ± 17.3 metabolites associated with 579.4 ± 30.6 genes. In the omnivore group, there are 924.5 ± 30.2 reactions and 812.8 ± 27.4 metabolites associated with 571.8 ± 23.0 genes. While in the sourdough group, there are 864.2 ± 39.0 reactions and 773.0 ± 14.3 metabolites associated with 500.5 ± 19.4 genes. The herbivore core metabolic model included 847 reactions and 771 metabolites, corresponding to 85.9 and 90.0% of the pan model. The omnivore core metabolic model included 796 reactions and 715 metabolites, corresponding to 80.73 and 83.82% of the pan model. And the sourdough core metabolic model included 752 reactions and 726 metabolites, corresponding to 81.91 and 91.20% of pan model.

Previous comparative genomic analysis showed that there are host-specific genes in different groups ([20] and Fig. 4b). However, slight differences in reactions and metabolites were observed between these strains and groups (Fig. 4c, d). In this comparison, only appearing in one group is considered specific, appearing in all groups is considered common and appearing in two (between one and all) groups is considered dispensable. As shown in Fig. 4b, the specific and common genes are 20.7 and 50.4% respectively. In our models (Fig. 4c, d., there are 7.8% specific reactions and 5.5% specific metabolites correspondingly 74.8% common reactions and 83.7% common metabolites. Here we noticed that the percentage of specific genes is more than specific model reactions and metabolites, while the common percentage is opposite, low correlation suggests that the many of the differences in the genome are not inherited to GEMs. Finally, we obtained core and pan models of the 35 strains, containing 671 reactions and 666 metabolites in the core model, while there are 1010 reactions and 870 metabolites in the pan model.

We compared the synthesis capacities of products between the three groups and the results are shown in Fig. 3. Most of the strains have similar carbohydrate metabolism pathways and have the capacity to produce acetate, ethanol and lactate. While for strain-dependent products like histamine and 1,3-propanediol, the synthesis pathways have differences both in and between groups. We find that the herbivore-derived *L. reuteri* maybe have the most completed metabolic pathways related to histamine and 1,3-propanediol because most models in herbivores could produce them (Fig. 3). However, all the GEMs in the sourdough group cannot produce histamine and 1,3-propanediol, this suggests that they may have less beneficial effects on their host. The omnivore group has the most differences within the group, i.e. there are 42.9 and 78.6% have the capacity to produce histamine and 1,3-propanediol separately. And the methylglyoxal synthase (*mgsA*) gene was missing in all GEMs of 35 strains, which explains why 1-propanol was not produced.

From the comparison of model characteristics and synthesis capacities, we found that the metabolism of the three groups of models is very similar, after all, there are more than 95% common reactions. However, the differences cannot be ignored, especially the ability to provide potentially beneficial metabolites. For instance, herbivore-derived *L. reuteri* may have some advantages in producing histamine and 1,3-propanediol, which provided the potential to be explored as a probiotic.

## Conclusions/ discussion

Here we reconstructed a GEM of *L. reuteri* ATCC PTA 6475 that can be used to simulate the metabolic capabilities and growth rates under different mediums. Most of GEM predictions were matched with experimental data except for the essential of arginine. Furthermore, core- and pan- GEMs of 35 *L. reuteri* strains were reconstructed and based on these we identified different synthesis capacities of histamine and 1,3-propanediol among these strains. These metabolic differences demonstrate some of the advantages of herbivore-derived *L. reuteri* which could provide potential assistance in the study of strain specificity and the exploration of future industrial strains. All the GEMs of *L.reuteri* provide a reliable basis to investigate the metabolism of *L. reuteri* in detail and their potential benefit on host health.

## Supplementary Information

**Additional file 1. **Model file of *i*HL622 and 35 pan-GEMs in SBML format.

**Additional file 2: Fig. S1.** Comparison of *L. reuteri* ATCC PTA 6475 sequences and COG functional distribution.

**Additional file 3: Table S1.** Information of 35 *L. reuteri* strains.

## Data Availability

All data generated or analyzed during this study are included in this published article and its supplementary information files.

## Abbreviations

GEM: Genome-scale metabolic model
FBA: Flux balance analysis
