# Supplementary material for: Genome-scale insights into the metabolic versatility of Limosilactobacillus reuteri
Source: BMC Biotechnol. 2021 Jul 30;21:46. doi: 10.1186/s12896-021-00702-w (PMC8325179; doi:10.1186/s12896-021-00702-w)

**Figure S1**

**Figure S1. Comparison of three version of *L. reuteri* ATCC PTA 6475 sequences and COG functional distribution.** (**a**) The COG functional distribution of genes in BioGaia genome J, translation, ribosomal structure and biogenesis; K, transcription; L, replication, recombination and repair; D, cell cycle control, cell division, chromosome partitioning; V, defense mechanisms; T, signal transduction mechanisms; M, cell wall/membrane/envelope biogenesis; N, cell motility; O, posttranslational modification, protein turnover, chaperones; C, energy production and conversion; G, carbohydrate transport and metabolism; E, amino acid transport and metabolism; F, nucleotide transport and metabolism; H, coenzyme transport and metabolism; I, lipid transport and metabolism; P, inorganic ion transport and metabolism; Q, secondary metabolites biosynthesis, transport and catabolism; R, general function prediction only; S, function unknown. *, no COG categories. (**b**) The venn diagram of common and unique genes in the three *L. reuteri* ATCC PTA 6475 sequences. V1 and V2 collected from the NCBI database with accession numbers of NZ_ACGX00000000 and GCF_000159475.2 separately. V3 sequenced from BioGaia.


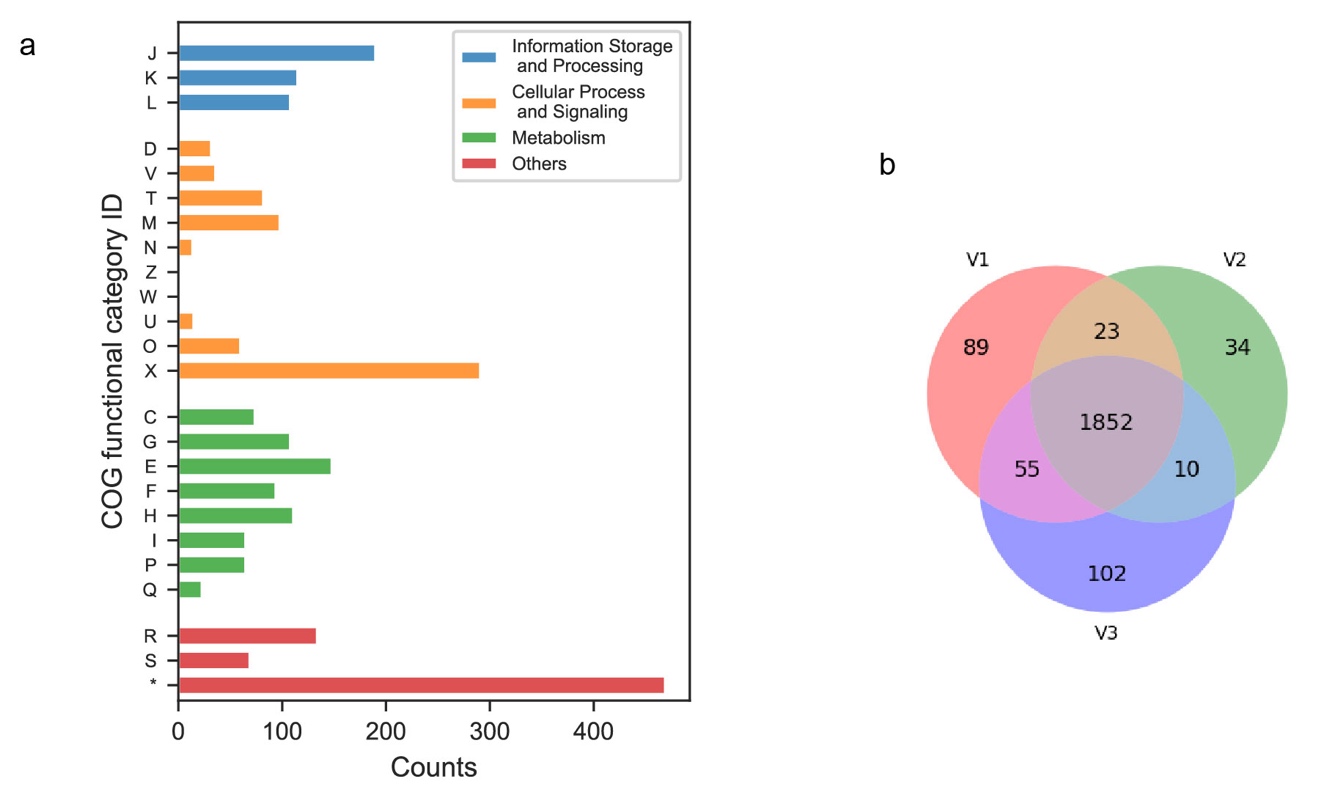

Supplement: Supplementary file 2 — Additional file 2: Fig. S1. Comparison of L. reuteri ATCC PTA 6475 sequences and COG functional distribution. [file 12896_2021_702_MOESM2_ESM.docx]
